# Impact of the COVID-19 Pandemic and Control Measures on Screening and Diagnoses of Type 2 Diabetes in British Columbia

**DOI:** 10.3390/ijerph22040519

**Published:** 2025-03-28

**Authors:** Bushra Mahmood, Gordon Li, Julia Li, James Wilton, Tricia S. Tang, Héctor Alexander Velásquez García, Stanley Wong, Akshay B. Jain, Zaeema Naveed, Arun Garg, Amandeep Nandra, Naveed Zafar Janjua, Geoffrey McKee

**Affiliations:** 1 Division of Endocrinology, Department of Medicine, University of British Columbia, Vancouver, BC V5Z 1M9, Canada; tricia.tang@vch.ca; 2 British Columbia Center for Disease Control (BCCDC), Vancouver, BC V5Z 4R4, Canada; julia.li@bccdc.ca (J.L.); james.wilton@bccdc.ca (J.W.); hector.velasquez@bccdc.ca (H.A.V.G.); stanley.wong@bccdc.ca (S.W.); zaeema.naveed@bccdc.ca (Z.N.); geoffrey.mckee@bccdc.ca (G.M.); 3 Provincial Health Services Authority, Vancouver, BC V6H 4C1, Canada; gordon.li@phsa.ca; 4 TLC Diabetes and Endocrinology, Surrey, BC V3T 0P8, Canada; oxyjain@gmail.com; 5 Fraser Health, Surrey, BC V3T 0H1, Canada; arun.garg@fraserhealth.ca; 6 British Columbia Cancer Agency, Surrey, BC V3V 0E8, Canada; 7 School of Population and Public Health, University of British Columbia, Vancouver, BC V6T 1Z3, Canada; 8 Center for Health Evaluation and Outcome Sciences (CHEOS), St. Paul’s Hospital, Vancouver, BC V6Z 1Y6, Canada

**Keywords:** type 2 diabetes, COVID-19, pandemic, control-measures, health service delivery

## Abstract

Introduction: In British Columbia (BC), Canada, COVID-19 and associated control measures impacted routine care for patients with diabetes. Some of these measures may have impacted timely screening and diagnosis of type 2 diabetes. We assessed the impact of control measures on screening and diagnosis of type 2 diabetes in BC. Methods: We used data from the BC COVID-19 Cohort, which includes COVID-19 and healthcare administrative data on all residents of BC. We assessed and compared screening (≥40 yrs) and diagnosis (≥18 yrs) of diabetes among the adult population during the pandemic period (1 April 2020–31 December 2022), with 1 January 2016–31 March 2020 used as a historical reference period. We used interrupted time series with generalized additive models to evaluate the impact of policy measures on screening and diagnoses trends. Results: We observed an initial decline in the mean number of screenings and diagnoses. In the third post-policy phase (January 2022–December 2022), there was a 4.8% (−5.1, 15.4) increase in screenings while after an initial reduction in diabetes diagnoses, we observed a significant increase of 31.6% (17.8, 46.6) in the third post-policy phase. Further stratification by age and sex showed the entire increase in diagnoses trends was driven by younger females with a 56.4% (25.1, 92.9) and 58.7% (38.2, 81.3) increment in diagnoses in the 18–29 and 40–49 age groups, respectively. Conclusions: The initial reduced number of screenings and diagnoses followed by the significant upward trend in diabetes diagnoses in the later post-policy phase have important clinical and public health implications. Further research is needed to understand the post-pandemic increase in diabetes among females.

## 1. Introduction

The COVID-19 pandemic and associated control measures led to disruption of medical care across Canada, including in the province of British Columbia (BC). On 17 March 2020, the BC Provincial Health Officer declared a public health emergency under the Emergency Programme Act to support the province-wide response to the novel coronavirus pandemic [1]. With this declaration, BC joined other Canadian provinces in rolling out control measures to prevent the transmission of severe acute respiratory syndrome coronavirus 2 (SARS-CoV-2), the virus that causes COVID-19, to reduce its impact on the health of the population. Several of these measures negatively impacted processes of routine care for individuals living with chronic disease [2]. Prevention and treatment services for non-communicable diseases across the country were disrupted due to either a shortage of staff or reallocation of resources [2]. To reduce the risk of SARS-CoV-2 transmission, many in-person visits to hospitals or clinics were either cancelled or replaced by virtual visits [3,4], while across BC, primary care, dental, pharmacy, and diagnostic services were most frequently reported as being difficult to access [5].

Although the disruptions in service delivery generally affected care pathways for all chronic illnesses, management of diabetes was particularly impacted [3,6,7]. Diabetes heightens the risk of severe complications like amputation, vision loss, and kidney failure. It is also linked to cardiovascular disease, dementia, certain cancers, and infections such as tuberculosis and severe COVID-19 [8]. However, timely detection of diabetes at the primary healthcare level, and treatment with oral hypoglycemic drugs or insulin, as well as newer injectable medications, can reduce the risk of complications and slow their progression [9]. Failure to treat or delays in timely diagnosis and treatment can lead to a higher risk of complications and even death.

According to one global World Health Organization (WHO) survey of 155 countries, 53% of the countries surveyed reported a partial or complete disruption of services for hypertension treatment; 49% for treatment for diabetes and diabetes-related complications [10]. In a survey of healthcare professionals from 47 countries across the world, diabetes (38%) was the condition reported to be most impacted by the reduction in healthcare resources due to COVID-19 [6]. Early studies from the United Kingdom (UK) highlighted the enormous impact of COVID-19 measures on diabetes diagnoses, routine testing, and diabetes-related complications [11,12,13]. It is estimated that in the province of BC—the third largest province in Canada with a population close to 5.4 million [14]—almost 19% of the population will be living with diabetes (diagnosed and undiagnosed) by 2032 with direct cost to the health system being close to $732 million [15]. Primary care-based interventions for the early detection and management of diabetes and diabetes-related complications are the cornerstone of comprehensive secondary prevention [16]. Diabetes Canada 2018 Guidelines recommend that in the absence of risk factors, all adults ≥40 years undergo screening for diabetes at least once every 3 years [17]. However, with restricted access to chronic disease care during the pandemic, it is not known how much routine screening and diagnosis of diabetes may have been impacted in BC. Our aim for this study was to assess the impact of the COVID-19 pandemic and related control measures on screening and diagnosis of type 2 diabetes among British Columbians.

## 2. Methods

Study setting and population: For this population-based cohort study, we compared and assessed changes in screening and diagnosis of type 2 diabetes in all adults living in BC. Screening individuals as early as aged 40 years old in primary care offices has proven to be useful in detecting unrecognized diabetes [18].

Inclusion criteria:

1. BC residents with a valid Personal Health Number (PHN). PHN is a unique identifier for British Columbia residents who are enrolled in the Medical Services Plan (MSP) and is used to access healthcare services;
2. BC adults ≥ 40 years (for screening) and BC adults ≥ 18 years (for type 2 diabetes diagnosis);
3. No prior diagnosis of diabetes.

Cases of gestational diabetes were excluded following CDR’s specifications. We also excluded people with type 1 diabetes based on any dispensation record for an insulin-related medication (Supplementary Materials File S2).

Canada has universal healthcare insurance, and all BC residents are covered for premium-free laboratory, physician, and hospital services by the province’s Medical Services Plan (MSP). The MSP claims database provides records of all healthcare services delivered by physicians to patients eligible for coverage. We compared monthly counts for screening and diagnosis of type 2 diabetes during 1 April 2020–31 December 2022 (post-policy period), with historical trends using 1 January 2016–31 March 2020 (pre-policy period) as our reference period.

### 2.1. Data Source

We conducted this study using linked provincial health data from the British Columbia COVID-19 Cohort (BCC19C). The BCC19C is a population-based surveillance platform, which integrates COVID-19 data (laboratory tests, case data, immunizations, and hospital and Intensive Care Unit (ICU) admissions), with healthcare utilization datasets. Healthcare utilization datasets and registries covering BC residents include medical visits (MSP billing claims), hospital admissions (Discharge Abstract Database), emergency room visits (National Ambulatory Care Reporting System), dispensed prescription drugs (PharmaNet), Chronic Disease Registry (CDR), and laboratory tests from the Provincial Laboratory Information System (PLIS) (Supplementary Materials File S1). PLIS captures approximately 95% of tests from private and public labs, including glycated hemoglobin (HbA1c), and other exams used in diabetes screening, diagnosis, and management (e.g., fasting plasma glucose (FPG)/or oral glucose tolerance test (75g.OGTT)/or random plasma glucose test (RPG)).

### 2.2. Study Exposure and Outcomes

As the emergency declarations in March 2020 represented a major shift in policy and likely coincided with a change in the public’s perceptions of the pandemic, we designed our study to model a pre-policy (pre-pandemic) phase starting January 2016 and ending in March 2020 and a post-policy (pandemic/post-pandemic) phase from April 2020 to December 2022. To capture the evolution of changes in screening and diagnoses of type 2 diabetes, the post-policy period was further categorized into three phases; April 2020–December 2020 (which coincided with the implementation of restrictive control measures in the absence of any intervention), January 2021–December 2021 (which coincided with a roll out of vaccination across BC and the re-imposition of a public health emergency), and January 2022–December 2022 (which coincided with the start of the highly infective Omicron variant wave and relaxation of some of the measures as the year proceeded) [19]. Our primary outcomes were a monthly number of diabetes screenings and diagnoses pre-pandemic in contrast with the pandemic period. Screening tests included either an FPG, an HbA1c, a 75g-OGTT, or RPG [17]. An individual was considered ‘screened’ if a fee item code was present for the measurement of an FPG, an HbA1c, a 75g-OGTT, or RPG.

We used a validated algorithm for diagnosis of diabetes based on medical visits, hospitalization records, CDR data, and prescription drug use for diabetes management (Supplementary Materials File S2) [20,21]. All cases identified based on antihyperglycemic prescription drugs (excluding insulin) that did not have a diabetes diagnostic code were investigated further to ensure these were true diabetes cases (Supplementary Materials File S2).

### 2.3. Study Covariates

Apart from age and sex, our covariates included BC’s five health authority regions (Fraser, Interior, Northern, Vancouver Coastal, and Vancouver Island), and urban/rural residence (based on BC’s Community Health Service Areas and classified as metropolitan, large urban, medium urban, small urban, rural hub, rural, and remote).

### 2.4. Statistical Analysis

The data were structured in a time-series format where screening and diagnoses were aggregated by year and month with stratification by sex, age group, urban/rural residence, and health authority region. We performed interrupted time series analysis using generalized additive models (GAMs), which has been demonstrated to perform well in modelling post-policy changes in outcomes in the presence of non-linearity [22]. Models used a log link function with a negative binomial distribution.

We used the regression models to compare the expected estimated monthly counts with counterfactual monthly counts after pandemic-related policy changes. To account for possible seasonality and long-term linear trends, calendar month and month since pandemic were fitted as continuous variables, the pandemic factor was fitted as binary variable. To account for systematic calendar-related variation (seasonal effects) associated with the month of December (when healthcare utilization is observed to decline over the holidays [23]), a variable was created that indicated if the calendar month was December or not and was added to fit the model in the screening data. The factor of ‘year’ as a categorical variable contributed to the modelling of diagnoses data. Eventually, the monthly expected rates and their 95% confidence intervals were plotted against the counterfactual counts to arrive at the absolute and relative difference between the estimated counts and counterfactual counts by Bayesian posterior probability.

In our sensitivity analysis, we further stratified diabetes screening and diagnoses trends by sex and age groups (40–49, 50–59, 60–69, 70–79, and >79 years for screening and 18–29, 30–39, 40–49, 50–59, 60–69, 70–79, and >79 years for diabetes diagnoses).

All data processing was conducted using MS SQL Server Management Studio 18 and statistical analyses were completed by R statistical software version 4.0.2 (R Project for Statistical Computing).

This study was approved by the University of British Columbia Behavioural Research Ethics Board (H20-02097).

## 3. Results

### 3.1. Diabetes Screening

At the start of 2016, there were 6,996,936 BC residents ≥ 40 years of age who were eligible for diabetes screening. By the end of the year, 930,046 (13%) had been screened according to Diabetes Canada Guidelines (Supplementary Table S1. The mean (*SD*) monthly number of screenings during the pre-policy period from January 2016–March 2020 were 79,045 (9199) compared to a mean of 78,717 (14,663) during the post-policy period from April 2020–December 2022. The mean number of screenings increased for females in the post-policy period. In the post-policy period, screening declined in the younger age groups, 40–49 and 50–59, compared to before. Metropolitan areas saw an increase in screening in the post-policy phase. The Interior, Northern, and Vancouver Coastal Health Authority region all experienced a decline in the mean number of screenings in the post-policy period (Table 1).

**Figure 1:**
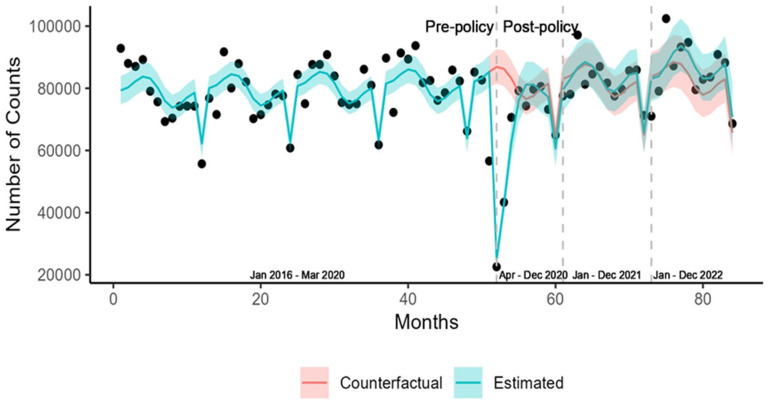
Observed and expected screening counts for type 2 diabetes in British Columbia.

We observed a steep decline in the number of screenings immediately following the declaration of a public health emergency and the initial implementation of pandemic measures in March 2020 (Figure 1).

Screening counts remained below expected during the first post-policy phase, with a gradual but statistically insignificant increase of 4.8% in the third post-policy phase, relative to the counterfactual or the expected. Stratification by sex, age, rural/urban residence, and Health Authority region did not suggest any statistically significant upward or downward trend in screening in the second post-policy phase except for the youngest age group (40–49), where screening remained at 9.8% (−17.0, −2.1) below expected. This downward trend among 40- to 49-year-olds extended to the third post-policy phase, where screening dropped significantly by 20% compared to the counterfactual (Table 2).

### 3.2. Diabetes Diagnoses

At the start of 2016, there were 9,488,922 adults (≥18 years old) living in BC. By the end of the year, 37,115 BC adults (54% females) had been diagnosed with diabetes (Supplementary Table S2). In the pre-policy phase, the mean (*SD*) number of individuals diagnosed with type 2 diabetes per month was 2705 (386). In the post-policy phase, there was a decrease to a mean of 2526 (367). In the post-policy phase, the mean number of diagnoses for the 30 to 39 and >79-year-old age groups increased (Table 3).

**Figure 2:**
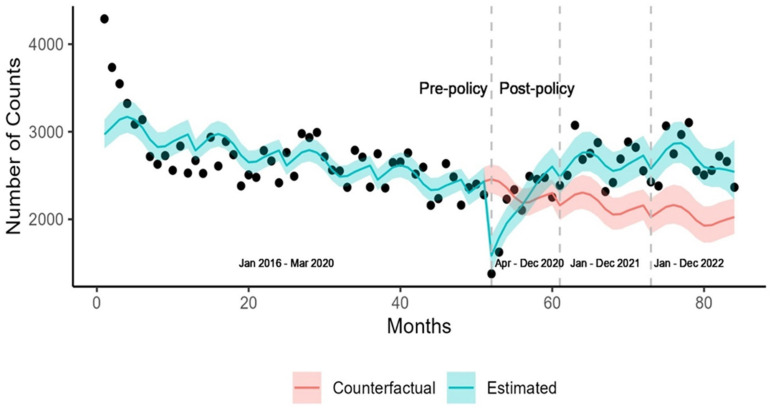
Observed and expected diagnosis counts for type 2 diabetes in British Columbia.

We observed an overall decline in the number of diabetes diagnoses following the initial implementation of pandemic measures (Figure 2).

After a 6.3% reduction in the first post-policy phase, a significant increase of 31.6% relative to the counterfactual was observed in the third post-policy phase. In the first post-policy phase, the only group that experienced a significant increase in diagnoses was the youngest age group (18–29) with a 23% (5.1, 41.8) increase in cases compared to the counterfactual. In the second post-policy phase, we observed a significant increase that was consistent across all groups except Vancouver Island Health Authority region. This significant increase in diagnoses continued in the third post-policy period. Diagnoses among females were 40.3% (25.2, 56.7) higher compared to the counterfactual (Table 4).

We noted a significant increase of 14.9% and 14.1% in diabetes diagnoses relative to expected in rural hub and rural regions, respectively. Stratification by Health Authority region showed a significant 59% (39.2, 80.6) increase in diabetes diagnoses in the Interior and a 31.8% (17.4, 47.4) increase from the expected in the Fraser Health Authority regions (Table 4).

Further stratification by sex and age revealed significant differences in the mean number/percent decrease in screenings for men in the age group 40–49 (Supplementary Table S3) and increase in diagnoses for younger females relative to the expected. In 2021, a significant 27% (7.7, 48.7) increase in diagnoses was observed for 18–29-year-old females relative to the counterfactual. This increased to 56.4% (25.1, 92.9) in the third post-policy phase (January 2022–December 2022). Compared to the counterfactual, we continued to observe a significant increase in diagnoses for females in the 30 to 39 (49.7% (29.4, 72.4)), 40 to 49 (58.7% (38.2, 81.3)) and 50- to 59-year-old (48.7% (31.5, 67.5)) age groups (Supplementary Table S4).

## 4. Discussion

We used population-based linked administrative data to investigate the impact of the COVID-19 pandemic and related control measures on screening and diagnoses of type 2 diabetes. Our analysis shows a significant overall decline in diabetes screening and diagnoses following the declaration of a public health emergency in BC and initial implementation of pandemic measures. However, there was evidence of an increase in screening and diabetes diagnoses in the third post-pandemic policy phase (January 2022–December 2022). These findings indicate that there was a significant negative impact on diabetes screening during the pandemic, particularly immediately following the initial implementation of control measures.

A comparison with pre-pandemic trends shows an 18% decline in screenings in the months following the initial imposition of policy measures. In the third post-policy phase, the observed screening trends appear to increase slightly relative to the expected (4.8%); however, this upward trend is not significant. We observed a steep decline in diabetes diagnoses in the first post-policy phase; in the third post-policy phase a significant increase of 31.6% relative to the ‘expected’ was observed. This upward trend was driven almost exclusively by females 59 years old and younger. The significant increase in diabetes diagnoses observed after an initial decline has important implications in terms of care and management of diabetes as this may translate into a substantial population level burden.

Data on the impact of the COVID-19 pandemic and its associated measures on screening and diagnosis of type 2 diabetes is limited. A UK-based study reported a 35.6% reduction in screening, which equated to 149,455 missed screening tests during the COVID-19 impact period (23 March 2020 to 30 September 2020) [24]. Our findings are consistent with those of Fazli et al., who reported a drop in screenings from 5.0% in March 2019 to 1.5% in March 2021, with the sharpest decline (~70%) between the observed and the expected rates occurring between February 2020 and April 2020 in Ontario, Canada [25]. Similarly, we observed the steepest decline in the first post-policy phase. As screening, diagnosis, and treatment for diabetes usually occurs in the primary care setting, the decline in screenings may partly be explained by an overall decrease in the number of in-person visits to General Practitioners, as shown by studies investigating the effects of COVID-19-related control measures on access to chronic disease care, including diabetes [4,12,26]. A systematic review involving 20 countries found consistent evidence of major reductions in the utilization of healthcare services during the pandemic period up to May 2020, compared with previous years [3]. Carr and colleagues reported a sharp fall (76–88%) in rates of performing health checks among those diagnosed with type 2 diabetes in English practices across the UK when compared with 10-year historical trends [12]. In BC, a population-based health survey reported increased barriers to accessing healthcare, with primary care being the most avoided type of healthcare service [5].

While an overall downward trend in screening was consistent across both males and females, the third post-policy phase saw a rebound in screening for females, while in males relative to the expected, screening trends were still in the negative by the second post-policy phase and improved only slightly in the third post-policy phase (2.1, 95% CI −7.0, 11.9). Some of this could be attributed to higher rates of use of primary care physician services among females compared to males. There is substantial evidence regarding gender patterns in healthcare utilization, whereby women (despite accounting for antenatal and reproductive health services) have higher healthcare utilization, especially in preventive care, compared to men [27,28,29,30]. A population-based study in Canada investigating disparities in adherence to diabetes screening guidelines among males and females found females had a significantly higher likelihood of being adherent than males [16]. The study also reported larger sex-differences in adherence in younger age groups than in older individuals aged 75–79 years [16]. While exploring healthcare utilization patterns were outside the scope of this study, documented evidence regarding higher engagement of women with healthcare services relative to men [16,27,31], especially preventive services among younger age groups, may explain why we see a stronger rebound in screening in the second and third post-policy phases among females compared to the counterfactual than in males.

Data on the impact of pandemic-related measures on the diagnosis of type 2 diabetes at a population level is limited. A UK-based study by Carr and colleagues noted a rate reduction of 70% (68–71%) in April 2020—immediately after control measures became legally effective in the UK towards the end of March [11]. Older individuals (i.e., ≥65 years), men, and people from deprived areas, had the greatest reductions in diagnosis rates [11]. A study from Salford, UK, observed 135 fewer diagnoses (a 49% reduction) of type 2 diabetes than expected between March and May 2020 [32]. A study from Alberta, Canada reported a 32% drop in the rate of incident diabetes during the COVID-19 pandemic (up to 31 March 2021), than pre-pandemic (6.98 per 1000 PY for the period 1 April 2018 to 16 March 2020) [33]. Our findings corroborate earlier studies as we noted a more pronounced decline in the first post-policy phase with 1328 (6.3%) fewer cases of diabetes relative to the expected. Similarly to Carr et al., we saw a steeper decline in the observed versus the expected number of cases in males and in older age groups (≥70 years old). The reduced number of diabetes diagnoses in older age groups can partly be explained by early evidence from epidemiological studies regarding an association of older age with more severe outcomes for COVID-19, which impacted the healthcare utilization rates among this high-risk population, discouraging many from engaging with GPs, as well as other outpatient and inpatient services, to avoid risk of infection [12,34,35,36].

As the pandemic progressed, the reduced diagnosis rates increased significantly in the second post-policy phase across most sub-groups. In the third post-policy phase, relative to the expected, we observed a significant increase in all sub-groups with some of the highest percent increases being among females (40%), as well as those in the 40 to 49 age bracket (38%), rural hub areas (51%), and the Interior Health Authority region (59%). Further stratification by sex and age showed that this increase was almost entirely driven by females in the younger age groups (56% among 18–29-year-old and 50% among 30–39-year-old).

Some of the spike in the second post-policy phase may be attributed to a backlog of cases that were missed right after imposition of control measures. However, it is difficult to explain what could be driving this significant upward trend in diagnoses all the way into the third post-policy phase. Our findings corroborate recent studies that have shown a somewhat similar increase in diabetes incident during and after the COVID-19 pandemic compared with pre-pandemic time periods in children, young adults, and older (>40) adults [37,38,39,40]. A longitudinal study following 234,956 participants for six years from 1 January 2017 to 31 December 2022 reported an increase of two and a half times in incidence rate of type 2 diabetes—4.85 (95% CI, 4.68–5.02) per 1000 person-years in the period 2017–2019, vs. 12.21 (95% CI, 11.94–12.48) per 1000 person-years in 2020–2022 [37]. More recently, a report by Diabetes UK showed an almost 40% increase in 5 years (between 2015 and 2017 and 2022–2023) in the number of people diagnosed with type 2 diabetes in the UK who were younger than 40 years old [41]. On a global level, according to data from the 10th edition of the Diabetes Atlas, prevalence estimates of diabetes among people aged 20–39 years increased from 2·9% (63 million people) in 2013 to 3·8% (260 million) in 2021 [42]. Thus, type 2 diabetes, typically a condition predominantly associated with middle-aged and older adults, has become increasingly prevalent in young populations. This has serious implications for population health as early onset of type 2 diabetes is associated with greater insulin resistance, a faster decline in β cell function, and earlier and more severe complications, leading to increased morbidity and mortality [43]. According to a recent US-based study, at aged 50, individuals with type 2 diabetes diagnosed at age 30 years, died on average 14 years earlier than those without diabetes. By contrast, on average, less than 2 years of life are lost when type 2 diabetes presents after 70 years of age [44].

Sex patterns in the prevalence of diagnosed and undiagnosed type 2 diabetes and prediabetes are well established [16,18]. Worldwide, an estimated 17.7 million more men than women have diabetes mellitus [45]. As stated earlier, sex differences in adherence to screening guidelines for diabetes are also well recognized [16]. Since screening and diagnostic tests for diabetes are the same, evidently a higher detection of cases in females can partly be explained by higher screening counts among females compared to males. Higher screening among females could be a function of higher healthcare utilization but we do not know the contextual factors driving this utilization.

Studies have found a significant association between female sex and long COVID-19 syndrome [46,47], a condition characterized by a wide range of acquired sequela/complications that begin or persist beyond 4 weeks from the onset of acute COVID-19 symptoms [48,49,50,51]. There is a possibility that some of these encounters could have been driven by a high number of females presenting with long COVID-19 symptoms. This, however, is a speculation and needs further investigation. Despite low counts of screening among males, population-based studies have shown that males have higher rates of prediabetes and diabetes compared to females [16,52,53]. Our findings highlight the need to create more awareness around diabetes screening among males as delays in lifestyle changes and timely treatment initialization for diabetes can have significant implications for overall health.

Changes in lifestyle behaviours pertaining to diet and physical activity during the pandemic may also partially explain a spike in diagnoses. Healthy lifestyle habits can prevent or delay and even remit type 2 diabetes and reduce complications [54]. Studies have highlighted the negative impact of COVID-19 lockdowns on diet and physical activity behaviours with negative outcomes being associated with an increase in sedentary time, weight gain, and poor mental and physical health [55,56,57]. Moreover, gender differences in mental health have been documented, with women being more seriously affected by mental and psychosomatic ill-health during COVID-19 lockdowns [58]. A Latin American study reported healthier eating habits as well as lower tobacco and alcohol consumption among women than men but less exercise and increased symptoms of depression compared to men [59]. There is a possibility that sustained stress and unhealthy lifestyle behaviours during the pandemic could also have potentially accelerated diabetes, especially among females already at high risk for type 2 diabetes or with undiagnosed prediabetes [60].

Furthermore, there is emerging evidence of a bidirectional association between diabetes and COVID-19. Hyperglycemia, associated with inflammation and weakened immunity against infections, was recognized as a significant risk factor for severe COVID-19 early in the pandemic [61]. More recently, an association of COVID-19 with hyperglycemia is being observed in patients with no prior history of diabetes [62,63,64,65]. A meta-analysis of nine studies with approximately 40 million participants found that the incidence of diabetes after COVID-19 was 15.53 (7.91–25.64) per 1000 person-years, and the relative risk of diabetes after SARS-CoV-2 infection was elevated (RR 1.62 [1.45–1.80]) [65]. The relative risk of type 2 diabetes was RR = 1.70 (1.32–2.19), compared to non-COVID-19 patients [65]. The relative risk of diabetes in sex groups was about 2 (males: RR = 2.08 [1.27–3.40]; females: RR = 1.99 [1.47–2.80]) [65]. A recent Canadian study of 629,935 individuals from the British Columbia COVID-19 Cohort reported an adjusted hazard ratio of 1.17 (95% CI 1.06–1.28) of incident diabetes, with higher risk in males (adjusted HR 1.22, 95% CI 1.06–1.40) [62]. SARS-CoV-2 has, thus, been associated with an elevated risk of type 2 diabetes, but it is unclear whether incident diabetes is persistent over time or differs in severity over time. A US-based study observed the excess burden of diabetes among individuals non-hospitalized for COVID-19 to persist at 12 months [66]. Given the significant proportion of the population infected with SARS-CoV-2, further research is critical to assess the risk and burden of diabetes in the post-acute phase of COVID-19 to inform care strategies [66].

### Strengths and Limitations

To our knowledge, this is the first population-based study in BC to investigate the impact of the COVID-19 pandemic and related control measures on screening and diagnosis of type 2 diabetes. This study used interrupted time series design on a large-scale, most up-to-date linked health administrative data to illustrate the changes in trends compared to the pre-pandemic period. However, our study does have some limitations. Our analysis was based on administrative data with inherent data quality issues that may lead to misclassification bias. Second, in the absence of race-based data, we were limited in our ability to investigate ethnicity-based disparities in diabetes screening and diagnoses trends. Lastly, due to significant missingness of the material deprivation index or ability to link to other measures of socioeconomic status, we were not able to explore socioeconomic status variation in outcomes.

## 5. Conclusions

This study has shown the profound indirect impact of the COVID-19 pandemic and its related measures on chronic disease management and the scale of disruptions to the timely screening and diagnosis of type 2 diabetes. While screening for type 2 diabetes still does not appear to have recovered fully to the pre-pandemic levels, we observed an increase in the diagnoses of diabetes in the post-pandemic period compared to the expected. Increased trends in diagnoses are being driven almost exclusively by relatively younger females. This is concerning as historically, epidemiological studies have shown a higher prevalence of prediabetes and diabetes in males despite suboptimal adherence to diabetes screening. Additionally, diabetes diagnoses at a younger age means a higher risk of developing micro- and macrovascular complications of diabetes much earlier and with more severe complications, leading to increased morbidity and mortality.

Our research findings have important implications and require a reprioritization of clinical guidelines and public health policies and targeted interventions for increased screening for timely diagnosis of diabetes. Our research also underscores the importance of adopting fresh perspectives and innovative work models in pandemic-like situations to effectively manage and prevent diabetes and its associated complications. Evidence-based interventions to reduce the risk of diabetes-related complications include controlling glycemic levels and blood pressure, managing lipids, quitting smoking, maintaining a healthy diet, engaging in regular exercise, and ensuring timely screening for renal, foot, and retinopathy complications [67]. A pandemic situation requires practitioners to assess what aspects of care must remain the same versus what can be effectively shifted to virtual care or deferred to a later date. People living with diabetes should be encouraged to enhance their self-management of the condition [15,16,62,63]. Additionally, advanced glucose monitoring systems and other technologies such as blood pressure monitors can support both individuals with diabetes and their healthcare teams in managing the condition more effectively. Lastly, diabetes professionals must be mindful of the individual differences among their patients. It is important to recognize who will thrive during times of uncertainty and reduced activity, and who will require extra support to maintain a healthy lifestyle and optimal glycemic management [67].

Further research is critical to understand underlying causes (other than a backlog) that may be driving this epidemic of diabetes—specifically among females. Additionally, future research is needed to understand how ethnicity and social deprivation might explain these differences so that targeted strategies and interventions can be developed to tackle these crises in the most vulnerable and high-risk population subgroups.

Chronic disease registry (CDR)—Available information includes diagnosis date(s)

- Medical Services Plan (MSP)—ICD-9 billing/diagnostic codes;
- Discharge Abstract Database (DAD)—DAD1 contains ICD-9 coded hospitalization data and DAD2 contains ICD-10 coded hospitalization data;
- National Ambulatory Care Reporting System (NACRS)—Contains ICD-10 coded diagnostic codes;
- PharmaNet—Each medication is identified with a drug identification number (DINPIN);
- Provincial Lab Information System (PLIS);
- Canadian Census (2016);
- Vital Statistics (VS);
- Client Roster: for sociodemographic information on each client.

All inferences, opinions, and conclusions drawn in this publication are those of the author(s), and do not reflect the opinions or policies of the data steward(s).

## Figures and Tables

**Table 1:** Monthly mean number of diabetes screenings for individuals ≥40 years of age during the pre-policy period (1 January 2016–31 March 2020) and the post-policy period (1 April 2020–31 December 2022).

Population	Number of Screenings (Pre-Policy)	Number of Screenings (Post-Policy)
	Mean (SD)	Mean (SD)
Total	79,045 (9199)	78,717 (14,663)
Sex		
Female	42,573 (5426)	42,650 (8396)
Male	36,472 (3943)	36,067 (6352)
Age Group		
40–49	12,193 (2082)	6846 (1373)
50–59	22,514 (2369)	21,679 (4106)
60–69	23,191 (2778)	23,597 (4728)
70–79	14,724 (2344)	17,777 (3522)
>79	6423 (1022)	7818 (1613)
Urban/Rural Residence		
Metropolitan	35,769 (3899)	36,401 (6884)
Large Urban	14,182 (1660)	13,988 (2480)
Medium Urban	8006 (1070)	7822 (1480)
Small Urban	7415 (958)	7241 (1401)
Rural Hub	4138 (546)	4010 (745)
Rural	8880 (1187)	8653 (1697)
Remote	630 (87)	596 (108)
Missing	24 (36)	6 (3)
Health Authority		
Fraser	26,608 (3000)	26,911 (5038)
Interior	13,371 (1875)	12,904 (2470)
Northern	4142 (510)	3997 (815)
Vancouver Coastal	19,557 (2130)	19,919 (3803)
Vancouver Island	15,343 (1868)	14,981 (2656)
Missing	24 (36)	6 (3)

**Table 2:** Difference in number of screenings following pandemic-related policies, compared to counterfactual, by period.

Population	Absolute Difference, n (95% CI)			Percentage Difference, % (95% CI)		
	1 April 2020–31 December 2020	1 January 2021–31 December 2021	1 January 2022–31 December 2022	1 April 2020–31 December 2020	1 January 2021–31 December 2021	1 January 2022–31 December 2022
Total	−129,346 (−180,717, −78,287)	−9381 (−72,360, 89,553)	44,690 (−55,288, 141,268)	−18.0 (−24.2, −11.4)	1.1 (−7.1, 9.7)	4.8 (−5.1, 15.4)
Sex						
Female	−65,620 (−95,029, −36,621)	18,785 (−27,965, 64,588)	35,325 (−21,114, 89,332)	−17.1 (−23.8, −10.0)	3.8 (−5.1, 13.2)	7.1 (−3.7, 18.7)
Male	−63,713 (−85,758, −41,726)	−9444 (−44,918, 25,495)	9106 (−34,765, 51,373)	−19.1 (−24.8, −13.0)	−2.0 (−9.3, 5.9)	2.1 (−7.0, 11.9)
Age Group						
40–49	−22,430 (−28,051, −16,907)	−10,094 (−18,319, −2039)	−18,632 (−27,313, −10,296)	−27.0 (−32.3, −21.2)	−9.8 (−17.0, −2.1)	−20.1 (−27.6, −12.0)
50–59	−41,887 (−55,558, −28,285)	3362 (−18,835, 25,003)	10,009 (−16,721, 35,717)	−20.8 (−26.7, −14.7)	1.3 (−6.6, 9.7)	3.9 (−5.8, 14.1)
60–69	−38,633 (−54,254, −23,014)	3293 (−21,640, 27,828)	16,964 (−14,148, 47,046)	−17.5 (−23.7, −10.9)	1.2 (−6.8, 9.7)	5.7 (−4.2, 16.3)
70–79	−24,228 (−36,296, −12,178)	−3856 (−23,741, 15,638)	3011 (−23,001, 27,643)	−15.6 (−22.6, −8.2)	−1.6 (−10.1, 7.5)	1.5 (−8.9, 12.7)
>79	−8353 (−13,716, −3016)	2754 (−6276, 11,545)	8881 (−2615, 19,989)	−12.9 (−20.5, −4.9)	3.1 (−6.3, 13.2)	9.3 (−2.5, 22.2)
Urban/Rural Residence						
Metropolitan	−59,800 (−83,955, −35,893)	6249 (−32,666, 44,466)	18,308 (−29,431, 63,990)	−18.1 (−23.4, −11.3)	1.5 (−6.8, 10.4)	4.2 (−5.8, 15.1)
Large Urban	−20,778 (−29,722, −11,913)	1466 (−12,722, 15,331)	8050 (−9212, 24,594)	−16.2 (−22.5, −9.7)	1.0 (−6.9, 9.4)	4.9 (−4.8, 15.4)
Medium Urban	−13,787 (−19,059, −8545)	−2406 (−10,905, 5865)	779 (−9545, 10,761)	−18.9 (−25.2, −12.2)	−2.3 (−10.3, 6.2)	0.9 (−8.8, 11.4)
Small Urban	−12,828 (−17,539, −8115)	1999 (−5592, 9447)	7112 (−2113, 16,046)	−19.6 (−25.8, −13.0)	2.3 (−6.0, 11.2)	8.2 (−2.2, 19.4)
Rural Hub	−6710 (−9456, −3953)	652 (−3758, 4978)	2327 (−3032, 7442)	−18.3 (−24.8, −11.3)	1.4 (−7.1, 10.5)	4.9 (−5.5, 16.2)
Rural	−14,922 (−20,644, 9236)	1048 (−8102, 10,017)	7275 (−3923, 18,049)	−19.0 (−25.3, −12.3)	1.1 (−7.2, 9.9)	7.0 (−3.4, 18.2)
Remote	−816 (−1292, −342)	−104 (−860, 627)	363 (−546, 1232)	−14.9 (−22.7, −6.5)	−1.2 (−10.7, 9)	5.3 (−6.7, 18.3)
Health Authority						
Fraser	−45,524 (−63,220, −28,008)	2527 (−26,147, 30,456)	11,864 (−22,867, 45,480)	−18.5 (−24.8, −11.9)	0.9 (7.4, 9.7)	3.7 (−6.3, 14.4)
Interior	−22,726 (−31,408, −14,063)	−666 (−14,579, 12,908)	7140 (−9774, 23,352)	−19.1 (−25.4, −12.3)	−0.3 (−8.5, 8.5)	4.7 (−5.5, 15.7)
Northern	−7585 (−10,171, −5023)	873 (−3355, 5008)	2867 (−2276, 7792)	−20.8 (−26.9, −14.3)	1.9 (−6.4, 10.6)	6.0 (−4.2, 16.9)
Vancouver Coastal	−31,049 (−44,310, −17,838)	5824 (−15,391, 26,724)	13,853 (−12,130, 38,875)	−17.3 (−23.8, −10.4)	2.5 (−6.0, 11.6)	5.8 (−4.6, 17.1)
Vancouver Island	−22,768 (−32,642, −12,950)	397 (−15,208, 15,675)	8412 (−10,411, 26,624)	−16.6 (−22.9, −9.9)	0.3 (−7.7, 8.8)	4.7 (−5.2, 15.4)

**Table 3:** Monthly mean number of individuals ≥18 years of age diagnosed with diabetes during the pre-policy period (1 January 2016–March 2020) and the post-policy period (1 April 2020–31 December 2022).

Population	Number of Individuals Diagnosed (Pre-Policy)	Number of Individuals Diagnosed (Post-Policy)
	Mean (SD)	Mean (SD)
Total	2705 (386)	2526 (367)
Sex		
Female	1269 (175)	1261 (203)
Male	1436 (219)	1266 (178)
Age Group		
18–29	66 (16)	61 (9)
30–39	212 (28)	223 (33)
40–49	433 (67)	406 (61)
50–59	719 (115)	640 (98)
60–69	718 (112)	637 (99)
70–79	400 (54)	397 (65)
>79	157 (20)	162 (27)
Urban/Rural Residence		
Metropolitan	1430 (195)	1330 (199)
Large Urban	430 (63)	416 (66)
Medium Urban	272 (41)	252 (39)
Small Urban	221 (43)	203 (35)
Rural Hub	110 (23)	100 (20)
Rural	218 (39)	206 (33)
Remote	18 (5)	17 (5)
Missing	6 (3)	3 (2)
Health Authority		
Fraser	1129 (159)	1062 (156)
Interior	370 (71)	344 (62)
Northern	152 (27)	149 (23)
Vancouver Coastal	633 (89)	592 (92)
Vancouver Island	414 (64)	376 (60)
Missing	6 (3)	3 (2)

**Table 4:** Difference in number of diabetes diagnoses following pandemic-related policies, compared to counterfactual, by period.

Population	Absolute Difference, n (95% CI)			Percentage Difference, % (95% CI)		
	1 April 2020–31 December 2020	1 January 2021–31 December 2021	1 January 2022–31 December 2022	1 April 2020–31 December 2020	1 January 2021–31 December 2021	1 January 2022–31 December 2022
Total	−1328 (−3034, 369)	5716 (3117, 8255)	7679 (4746, 10519)	−6.3 (−14.1, 1.7)	22.0 (11.2, 33.6)	31.6 (17.8, 46.6)
Sex						
Female	−426 (−1272, 423)	3451 (2164, 4726)	4658 (3185, 6098)	−4.2 (−12.4, 4.4)	28.1 (16.4, 40.7)	40.3 (25.2, 56.7)
Male	−917 (−1815, −19)	2267 (881, 3621)	3010 (1462, 4512)	−8.4 (−16.1, −0.1)	16.7 (6.1, 28.0)	23.8 (10.4, 38.3)
Age Group						
18–29	103 (26, 181)	163 (66, 254)	190 (73, 300)	22.5 (5.1, 41.8)	29.2 (10.3, 50.4)	37.8 (12.2, 67.4)
30–39	26 (−145, 196)	602 (348, 851)	695 (404, 977)	1.6 (−7.9, 11.9)	27.6 (14.7, 41.7)	33.3 (17.2, 50.9)
40–49	−115 (−410, 177)	1103 (662, 1537)	1413 (917, 1894)	−3.5 (−12.1, 5.7)	27.5 (15.3, 40.7)	38.0 (22.3, 55.2)
50–59	−460 (−924, −1)	1518 (817, 2206)	2055 (1275, 2811)	−8.6 (−16.6, −0.1)	23.2 (11.7, 35.7)	34.0 (19.1, 50.2)
60–69	−438 (−921, 45)	1336 (604, 2052)	1705 (892, 2487)	−8.0 (−16.3, 0.9)	20.1 (8.4, 32.6)	27.5 (13.0, 43.5)
70–79	−319 (−629, −14)	724 (246, 1191)	1136 (584, 1672)	−9.6 (−18.3, −0.4)	17.4 (5.5, 30.2)	28.4 (13.0, 45.3)
>79	−150 (−286, −16)	227 (17, 433)	381 (128, 622)	−11.0 (−20.0, −1.2)	13.1 (0.9 (26.3)	22.2 (6.7, 39.5)
Urban/Rural Residence						
Metropolitan	−837 (−1793, 114)	3208 (1760, 4635)	3664 (2039, 5248)	−7.5 (−15.5, 1.0)	23.4 (11.8, 35.9)	28.6 (14.3, 44.1)
Large Urban	−138 (−433, 160)	751 (306, 1190)	1340 (824, 1844)	−4.0 (−12.4, 5.0)	17.6 (6.6, 29.5)	33.0 (18.3, 49.2)
Medium Urban	−156 (−371, 59)	376 (54, 693)	567 (195, 923)	−7.2 (−16.5, 2.8)	14.0 (1.9, 27.1)	22.1 (6.7, 39.1)
Small Urban	−162 (−348, 24)	480 (194, 760)	780 (451, 1097)	−9.7 (−20.1, 1.5)	23.7 (8.5, 40.3)	41.4 (21.0, 64.4)
Rural Hub	−25 (−128, 79)	353 (201, 502)	422 (254, 583)	−3.0 (−15.6, 10.9)	38.4 (19.5, 59.3)	51.0 (26.6, 78.8)
Rural	−79 (−242, 86)	545 (298, 787)	790 (509, 1062)	−4.7 (−14.2, 5.6)	27.0 (13.5, 41.6)	42.1 (24.2, 61.6)
Remote	13 (−18, 44)	63 (20, 104)	64 (15, 109)	10.8 (−12.1, 37.9)	40.9 (10.5, 77.2)	46.0 (8.5, 92.5)
Health Authority						
Fraser	−486 (−1236, 261)	2590 (1455, 3706)	3209 (1931, 4448)	−5.5 (−13.6, 3.2)	24.1 (12.5, 36.4)	31.8 (17.4, 47.4)
Interior	−133 (−393, 127)	1082 (690, 1469)	1706 (1262, 2139)	−5.0 (−14.3, 5.1)	34.0 (19.9, 49.2)	59.0 (39.2, 80.6)
Northern	−40 (−184, 103)	284 (67, 498)	333 (82, 573)	−3.1 (−14.2, 9.0)	18.2 (3.7, 34.0)	22.3 (4.7, 41.9)
Vancouver Coastal	−327 (−784, 131)	1527 (829, 2213)	1499 (724, 2249)	−6.6 (−15.3, 2.7)	25.1 (12.5, 38.7)	26.3 (11.3, 42.7)
Vancouver Island	−393 (−716, −74)	260 (−235, 742)	866 (279, 1427)	−11.8 (−20.6, −2.3)	6.3 (−5.0, 18.6)	21.5 (6.2, 38.5)

## Data Availability

This study is based on data contained in various provincial registries and database. Access to the data could be requested through the BC Centre for Disease Control Institutional Data Access for researchers who meet the criteria for access to confidential data. Request for data may be sent to datarequest@bccdc.ca.

## Supplementary Materials

The following supporting information can be downloaded at: https://www.mdpi.com/article/10.3390/ijerph22040519/s1, Table S1: Total number of BC adults (≥40 years) eligible for screening and screened in 2016. Table S2: Total number of BC adults ≥ 18 years diagnosed with diabetes in 2016. Table S3: Difference in number of diabetes screening following pandemic-related policies, compared to counterfactual, by period, age group and sex. Table S4: Difference in number of diabetes diagnoses following pandemic-related policies, compared to counterfactual, by period, age group and sex. Figure S1: Diabetes diagnoses males (all ages). Figure S2: Diabetes diagnoses males (18–29 years). Figure S3: Diabetes diagnoses males (30–39 years). Figure S4: Diabetes diagnoses males (40–49 years). Figure S5: Diabetes diagnoses males (50–59 years). Figure S6: Diabetes diagnoses females (all age groups). Figure S7: Diabetes diagnoses females (18–29 years). Figure S8: Diabetes diagnoses females (30–39 years). Figure S9: Diabetes diagnoses females (40–49 years). Figure S10: Diabetes diagnoses females (50–59 years). References [17,20,21,68,69] are cited in the File S2.
