# Supplementary material for: Impact of the COVID-19 Pandemic and Control Measures on Screening and Diagnoses of Type 2 Diabetes in British Columbia
Source: Int J Environ Res Public Health. 2025 Mar 28;22(4):519. doi: 10.3390/ijerph22040519 (PMC12026491; doi:10.3390/ijerph22040519)
Supplement: Supplementary file 1 [file ijerph-22-00519-s001.zip › File S1_Revised.pdf]

File S1

Table 1. Data Sets Integrated Within British Columbia COVID-19 Cohort (BCC19C)

**British Columbia Centre for Disease Control (BCCDC), Provincial Heath Services Authority (PHSA) and Regional Health Authority data sources:**	**Data Date Ranges:**
Integrated COVID-19 laboratory dataset (SARS-CoV2 tests from private/public labs, includes sequencing and screening data) [S1]	Jan 2020-onward
COVID-19 surveillance case data (information collected on all probable/confirmed cases as part of public health follow up) [S2]	Jan 2020-onward
Provincial COVID-19 Monitoring Solution (critical and non-critical care hospital census data) [S3]	Jan 2020-onward
Provincial Immunizations Registry (COVID-19 vaccination data) [S4]	Dec 2020-onward
Provincial Laboratory Information Solution (laboratory tests from private/public labs) [S5]	Jan 2020-onward
Public Health Reporting Data warehouse (Influenza laboratory tests) [S6]	Jan 2008-onward
Emergency department visits (hospital-based and community-based ambulatory care)	Mar 2020-onward
**Ministry of Health (MoH) Administrative Data Sources:**	**Data Date Ranges:**
Client Roster (CR) (registry of enrollment in the universal public health insurance plan including residential history) [S7]	2008/9-onward
Discharge Abstracts Database (DAD) (hospital discharge records) [S8]	2008/9-onward
Medical Services Plan (MSP) (physician diagnostic and billing data for services provided through universal public health insurance plan) [S9]	2008/9-onward
PharmaNet (Pharma) (prescription drugs dispensed from community pharmacies, includes medications covered by public and private insurance plans) [S10]	2008/9-onward
BC Vital Statistics (VS) (deaths registry) [S11]	2008/9-onward
National Ambulatory Care Reporting System (NACRS) (hospital-based and community-based ambulatory care) [S12]	2011/12–onward
Chronic Disease Registry [S13]	2008/9-2018/19
811 Calls (respiratory calls only) [S14]	2014-onward
Health System Matrix [S15]	2018/19-onward
Population Grouper Methodology [S16]	2008/9-onward

S1. British Columbia Centre for Disease Control [creator]. Integrated COVID-19 laboratory dataset (SARS-CoV2 tests from private/public labs). Public Health Reporting Data Warehouse, British Columbia Centre for Disease Control [publisher] (2021). 2022.

S2. British Columbia Centre for Disease Control [creator]. COVID-19 surveillance case data. British Columbia Centre for Disease Control [publisher]. (2021). 2022.

S3. Provincial Health Services Authority [creator]. Provincial COVID-19 Monitoring Solution. Provincial Health Services Authority [publisher]. (2021). 2022.

S4. Provincial Health Services Authority [creator]. Provincial Public Health Information Systems [publisher]. (2021). 2022.

S5. Provincial Health Services Authority [creator]. COVID-19 vaccination data. Provincial Immunizations Registry, Provincial Public Health Information Systems [publisher]. (2021). 2022.

S6. British Columbia Centre for Disease Control [creator]. Respiratory datamart, Public Health Reporting Data Warehouse, British Columbia Centre for Disease Control [publisher] (2021). 2022.

S7. British Columbia Ministry of Health [creator]. Client Roster (Client Registry System/Enterprise Master Patient Index). British Columbia Ministry of Health [publisher]. Data Extract. MOH (2021). 2022. https://www2.gov.bc.ca/gov/content/health/health-forms/online-services

S8. British Columbia Ministry of Health [creator]. Discharge Abstract Database (Hospital Separations). British Columbia Ministry of Health [publisher]. Data Extract. MOH (2021). 2022. https://www2.gov.bc.ca/gov/content/health/health-forms/online-services

S9. British Columbia Ministry of Health [creator]. Medical Services Plan (MSP) Payment Information File. British Columbia Ministry of Health [publisher]. Data Extract. MOH (2021). https://www2.gov.bc.ca/gov/content/health/health-forms/online-services

S10. British Columbia Ministry of Health [creator]. PharmaNet. British Columbia Ministry of Health [publisher]. Data Extract. MOH (2021). 2022. https://www2.gov.bc.ca/gov/content/health/health-forms/online-services

S11. BC Vital Statistics Agency [creator]. Vital Statistics Deaths. BC Vital Statistics Agency [publisher]. Data Extract. BC Vital Statistics Agency (2021). 2022. https://www2.gov.bc.ca/gov/content/health/health-forms/online-services

S12. British Columbia Ministry of Health [creator]. National Ambulatory Care Reporting System. British Columbia Ministry of Health [publisher]. Data Extract. MOH (2021). 2022. https://www2.gov.bc.ca/gov/content/health/health-forms/online-services

S13. British Columbia Ministry of Health [creator]. Chronic Disease Registry. British Columbia Ministry of Health [publisher]. Data Extract. MOH (2021). 2022. https://www2.gov.bc.ca/gov/content/health/health-forms/online-services

S14. British Columbia Ministry of Health [creator]. 811 calls. British Columbia Ministry of Health [publisher]. Data Extract. MOH (2021). 2022. https://www2.gov.bc.ca/gov/content/health/health-forms/online-services

S15. British Columbia Ministry of Health [creator]. Health System Matrix. British Columbia Ministry of Health [publisher]. Data Extract. MOH (2021). 2022. https://www2.gov.bc.ca/gov/content/health/health-forms/online-services

S16. British Columbia Ministry of Health [creator]. Population Grouper Methodology. British Columbia Ministry of Health [publisher]. Data Extract. MOH (2021). 2022. https://www2.gov.bc.ca/gov/content/health/health-forms/online-services
