# Supplementary material for: Impact of the COVID-19 Pandemic and Control Measures on Screening and Diagnoses of Type 2 Diabetes in British Columbia
Source: Int J Environ Res Public Health. 2025 Mar 28;22(4):519. doi: 10.3390/ijerph22040519 (PMC12026491; doi:10.3390/ijerph22040519)
Supplement: Supplementary file 1 [file ijerph-22-00519-s001.zip › File S2_Revised.pdf]

File S2

Definitions for variables derived from administrative datasets

Administrative data sources:

A) Chronic disease registry (CDR) – Available information includes diagnosis date(s)
B) Medical Services Plan (MSP) – ICD-9 billing/diagnostic codes.
C) Discharge Abstract Database (DAD) – DAD1 contains ICD-9 coded hospitalization data and DAD2 contains ICD-10 coded hospitalization data.
D) National Ambulatory Care Reporting System (NACRS) – Contains ICD-10 coded diagnostic codes.
E) PharmaNet – Each medication is identified with a drug identification number (DINPIN).
F) Provincial Lab Information System (PLIS)
G) Canadian Census (2016)
H) Client Roster: for sociodemographic information on each client.

1) **Variable : Diabetes Mellitus (DM)**

Data source :
• Discharge Abstract Database (DAD)
• Medical Services Plan (MSP)
• Health Authority emergency department data (NACRS-HA)
• National Ambulatory Care Reporting System -Ministry of Health (NACRS-MOH)
• PharmaNet
• Vital Statistics (VS)
• Provincial Lab Information System (PLIS)

Definition :

Two or more physician visits with a diabetes MSP ICD 9/10 codes within two years; or two or more insulin prescriptions within one year; or one or more DAD code; or ne MSP fee item code; or one NACRS code; or one VS code; or one insulin and one oral antihyperglycemic (including metformin) prescription within 1 year; or two metformin prescriptions and 1 physician visit (MSP diagnostic code starting with 250) within 1 year; or two or more antihyperglycemic (not including metformin) prescriptions within 1 year.

Due to changes in indications for certain anti-hyperglycemic drugs like SGLT2 inhibitors and GLP1 Receptor Agonists for heart failure and CKD in people with or without diabetes, we reassessed and refined our diabetes case identification algorithm to increase sensitivity.

Diagnoses based on prescription dispensation of antihyperglycemic drugs other than insulin (in PharmaNet) were verified as true diabetes cases only if:

- ICD diagnostic codes for type 2 diabetes were found (MSP, DAD, NACRS, VS) (+/- 1 year of PharmaNET diagnosis; or
- Abnormal values for glucose tests in PLIS (+/- 1 year of PharmaNET diagnosis) were found; or
- History of glucose test strips dispensation (1 month before PharmaNET diagnosis or “forever” after PNET diagnosis) was found

Physician Billing Data: MSP ICD-9 diagnostic codes: starting with 249, 250; Fee items: exact codes: 585 14050 14250 33255 33256 96150

Hospitalization Data: DAD1/DAD3/ICD-9-CM diagnostic codes: starting with 249, 250

DAD2/NACRS/VS/ICD- 10-CA diagnostic codes: starting with E10 E11 E12 E13 E14 Y423 Z794 Z7984; DAD2 procedure codes: exact codes 1ZZ35CAB1 1ZZ35HAB1 1ZZ35YAB1

PNET dispensation codes:

5894, 6009, 12556, 12564, 12599, 12602, 12610, 13730, 13889, 15598, 21350, 21849, 24708, 24716, 93033, 156663, 156728, 178543, 209872, 209937, 244449, 274119, 274127, 275409, 275417, 275425, 312711, 312762, 314552, 377937, 399302, 420336, 430986, 431168, 446564, 446572, 446580, 446599, 446602, 446610, 454753, 480290, 480304, 513644, 514535, 514551, 539201, 539244, 542911, 542938, 542946, 546348, 552259, 552267, 552275, 554820, 586714, 586773, 587737, 612162, 612170, 612189, 612197, 612200, 612219, 612227, 612235, 612243, 612251, 612278, 612359, 614416, 628301, 632651, 632678, 632686, 632694, 644358, 646148, 648094, 650935, 720933, 720941, 723789, 733075, 765996, 773654, 795879, 808733, 808741, 889091, 889105, 889113, 889121, 999717, 1900927, 1900935, 1913654, 1913662, 1913670, 1913689, 1934066, 1934074, 1934082, 1934090, 1934104, 1934112, 1959212, 1959220, 1959239, 1959352, 1959360, 1962639, 1962647, 1962655, 1962663, 1985930, 1985949, 1985957, 1985965, 1985973, 1985981, 1986085, 1986791, 1986805, 1986813, 1986821, 1987534, 1987542, 1987828, 1987836, 2020734, 2020742, 2022230, 2022249, 2024217, 2024225, 2024233, 2024241, 2024268, 2024276, 2024284, 2024292, 2024306, 2024314, 2024322, 2024403, 2024446, 2025248, 2025256, 2045710, 2084341, 2085887, 2099233, 2147521, 2147548, 2148765, 2155850, 2162822, 2162849, 2167786, 2188902, 2190885, 2190893, 2220628, 2223562, 2224550, 2224569, 2224771, 2224798, 2226804, 2226812, 2228920, 2228939, 2229516, 2229517, 2229519, 2229595, 2229596, 2229656, 2229704, 2229705, 2229785, 2229994, 2230026, 2230027, 2230036, 2230037, 2230443, 2230444, 2230475, 2230670, 2230671, 2231058, 2231095, 2231096, 2231389, 2233562, 2233999, 2234513, 2234514, 2236543, 2236548, 2236733, 2236734, 2236985, 2236986, 2237531, 2238103, 2238469, 2238470, 2238471, 2238698, 2238827, 2239081, 2239214, 2239474, 2239475, 2239476, 2239924, 2239925, 2239926, 2240294, 2240295, 2240297, 2241111, 2241112, 2241113, 2241114, 2241283, 2241310, 2242095, 2242096, 2242572, 2242573, 2242574, 2242589, 2242726, 2242783, 2242793, 2242794, 2242931, 2242974, 2242987, 2244353, 2245247, 2245272, 2245273, 2245274, 2245397, 2245438, 2245439, 2245440, 2245689, 2246820, 2246821, 2246964, 2246965, 2247085, 2247086, 2247087, 2248008, 2248009, 2248210, 2248440, 2248441, 2248453, 2251930, 2252945, 2252953, 2254719, 2257726, 2257734, 2258781, 2258803, 2258811, 2265435, 2265443, 2265575, 2265583, 2268493, 2268507, 2269031, 2269058, 2269589, 2269597, 2269600, 2269619, 2271842, 2273101, 2273128, 2273136, 2273756, 2273764, 2273772, 2274248, 2274256, 2274264, 2274272, 2274914, 2274922, 2274930, 2275864, 2275872, 2276410, 2279061, 2279088, 2279126, 2279460, 2279479, 2279487, 2284545, 2284553, 2284782, 2284790, 2287072, 2294338, 2294346, 2294400, 2295377, 2295385, 2295393, 2297795, 2297906, 2297914, 2297922, 2298279, 2298287, 2298295, 2300451, 2301423, 2301431, 2301458, 2302861, 2302888, 2302896, 2302942, 2302950, 2302977, 2303124, 2303132, 2303140, 2303442, 2303450, 2303469, 2303922, 2305062, 2306166, 2306174, 2306182, 2307170, 2307189, 2307197, 2307553, 2307561, 2307588, 2307634, 2307642, 2307650, 2307669, 2307677, 2307723, 2312050, 2312069, 2312077, 2313596, 2314894, 2314908, 2316544, 2320754, 2320762, 2320770, 2321475, 2321483, 2321491, 2326329, 2326337, 2326345, 2326477, 2326485, 2326493, 2331519, 2331527, 2333554, 2333856, 2333864, 2333872,

2334437, 2334445, 2336316, 2339110, 2339129, 2339587, 2339595, 2340763, 2340771, 2341522, 2341603, 2343606, 2343614, 2345366, 2345374, 2345382, 2345854, 2345862, 2348578, 2350459, 2350467, 2351056, 2351064, 2353377, 2353385, 2354144, 2354152, 2354160, 2354349, 2354357, 2354365, 2354926, 2354934, 2354942, 2355663, 2355671, 2355698, 2356422, 2357453, 2357461, 2357488, 2357887, 2357895, 2357909, 2357917, 2357925, 2361264, 2361272, 2361809, 2361817, 2363232, 2363240, 2363259, 2363518, 2363704, 2363712, 2364506, 2364514, 2365286, 2365294, 2365529, 2365537, 2366347, 2366355, 2366363, 2370921, 2373270, 2373289, 2373297, 2374013, 2374021, 2374048, 2374587, 2374595, 2375842, 2375850, 2375869, 2375877, 2377209, 2378043, 2378051, 2378116, 2378124, 2378620, 2378639, 2378841, 2378868, 2379767, 2379775, 2380196, 2380218, 2380722, 2380730, 2384906, 2384914, 2384922, 2385341, 2385368, 2388766, 2388774, 2388839, 2388847, 2389169, 2389177, 2389185, 2389290, 2389304, 2389312, 2391600, 2397307, 2403250, 2403269, 2403277, 2403366, 2403374, 2403382, 2403412, 2403420, 2403439, 2403447, 2405067, 2406020, 2406039, 2407124, 2408228, 2408236, 2409283, 2409291, 2412829, 2415089, 2415968, 2415976, 2415984, 2416786, 2416794, 2416808, 2417049, 2417057, 2417065, 2417189, 2417197, 2417200, 2417219, 2417227, 2417235, 2418002, 2418010, 2418029, 2419300, 2419319, 2419327, 2419335, 2419343, 2419351, 2421674, 2421682, 2421690, 2421828, 2421836, 2423286, 2423294, 2424258, 2424266, 2424274, 2425483, 2425491, 2429764, 2429772, 2434121, 2434148, 2434156, 2435462, 2435470, 2437899, 2438275, 2438283, 2438658, 2439328, 2439611, 2441829, 2443635, 2443643, 2443937, 2443945, 2444844, 2444852, 2444933, 2444941, 2446065, 2448599, 2448602, 2448610, 2449390, 2449404, 2449765, 2449935, 2449943, 2455404, 2455412, 2455420, 2455439, 2455447, 2455455, 2456575, 2456583, 2456591, 2456605, 2456613, 2456621, 2456966, 2456974, 2459183, 2459752, 2459760, 2460408, 2460416, 2460424, 2460653, 2461323, 2461331, 2461528, 2463571, 2463601, 2463628, 2464276, 2464284, 2464349, 2466864, 2467879, 2467887, 2468603, 2468611, 2469871, 2469898, 2469901, 2470152, 2471469, 2471477, 2474875, 2475510, 2475529, 2475901, 2475928, 2476215, 2476223, 2476231, 2476258, 2477394, 2477408, 2477416, 2477424, 2478293, 2479575, 2479583, 2485664, 2492415, 2493373, 2493780, 2493799, 2494078, 2494086, 2494418, 2494442, 2497581, 2497603, 2497611, 2506564, 2506572, 2507471, 2507498, 2520303, 2520311, 22123367, 22303140, 44123021, 44123024, 44123025, 44123026, 44123028, 44123029, 44123033, 44123034, 44123035, 44123036, 44123037, 44123038, 44123040, 44123042, 44123043, 44123044, 44123045, 44123046, 44123047, 44123048, 44123049, 44123051, 44123052, 44123053, 44123055, 44123056, 44123057, 44123058, 44123059, 44123060, 44123061, 44123062, 44123063, 44123064, 44123065, 45230001, 45230002, 45230003, 45230004, 45230005, 45230006, 45230007, 45230008, 45230009, 45230010, 45230011, 45230012, 45230013, 45230014, 45230015, 45230016, 45230017, 45230018, 45230019, 46340034, 46340035, 46340036, 46340037, 46340038, 46340039, 46340040, 47450001, 47450002, 47450003, 47450004, 47450005, 47450006, 47450007, 47450008, 47450009, 48123021, 48123024, 48123025, 48123026, 48123028, 48123029, 48123033, 48123034, 48123035, 48123036, 48123037, 48123038, 48123040, 48123042, 48123043, 48123044, 48123045, 48123046, 48123047, 48123048, 48123049, 48123051, 48123052, 48123053, 48123055, 48123056, 48123057, 48123058, 48123059, 48123060, 48123061, 48123062, 48123063, 48123064, 48123065, 66123203, 66124134, 66124135, 66124215, 66124225, 66124232, 66124582, 66127961, 2052474, 2246613, 2246614, 2280418, 2405202, 2416239, 2428261, 2428288, 2428296, 2435772, 2435780, 2435799, 2449323, 2449331, 2459175, 2467860, 2483300, 2483319, 2503840, 2503859, 2503867, 2503956, 2503964, 2503972, 2504049, 2504057, 2504065, 2506270, 2506289, 2506297, 2508656, 2508664, 2508672, 2509415, 2509423, 2509431, 2512475, 2512483, 2512491, 2513331,

2513358, 2513366, 2513374, 2514486, 2514494, 2518732, 2518740, 2519852, 2519860, 2520184, 2520192, 2520206, 2520214, 2520222, 2520230, 2520494, 2520508, 2520516, 2520591, 2520605, 2520974, 2520982, 2522551, 2522578, 2522586, 2522594, 2522608, 2522705, 2522713, 2522721, 2523590, 2523604, 2523930, 2524333, 2524341, 2526441, 2526719, 2526727, 2526735, 2527189, 2527197, 2527650, 2527669, 2528509, 2528517, 2528525, 2528533, 2528541, 2528975, 2528983, 2529033, 2529041, 2529068, 2529106, 2529114, 2529122, 2529157, 2529165, 2529173, 2529254, 2529602, 2529866, 2529874, 2529882, 2531364, 2531372, 2531402, 2531410, 2531550, 2531569, 2531631, 2531658, 2531666, 2531895, 2531909, 2532654, 2532662, 2533049, 2533057, 2533073, 2533081, 2534134, 2534142, 2534150, 2534223, 2534231, 2534258, 2534290, 2534304, 2534312, 2534673, 2534983, 2534991, 2535009, 2535238, 2535297, 2535300, 2535513, 2535521, 2535548, 2535866, 2535874, 2536153, 2536161, 2536439, 2536447, 2536803, 2536811, 2536838, 2537656, 2537664, 2538334, 2538342, 2540118, 2540126, 2540134, 2540258, 2543117, 2543125, 2543133, 2543184, 2543192, 2543400, 2544423, 2544431, 2544458, 2532891, 2532905, 2532913, 2532921, 2532948, 2532956, 2541041, 2541068, 2541076, 2541084, 2541092, 2541106

Gestational Diabetes Exclusion codes:

Excluded gestational diabetes following CDRs specifications: Cases of suspected gestational diabetes in women aged 10-54 are not included by excluding hospitalizations, physician claims or prescriptions within the time period 120 days preceding or 180 days after hospital records containing birth-related diagnostic codes (see gestational diabetes exclusion codes below):

641,642,643,644,645,646,647,648,650,651,652,653,654,655,656,657,658,659,660,661,662,663,664,665 666,667,668,669,670,671,672,673,674,675,676,763,V27,O10,O11,O12,O13,O14,O15,O16,O21,O22,O23 ,O24,O25,O26,O28,O29,O30,O31,O32,O33,O34,O35,O36,O37,O40,O41,O42,O43,O44,O45,O46,O47,O4 8,O60,O61,O62,O63,O64,O65,O66,O67,O68,O69,O70,O71,O72,O73,O74,O75,O85,O86,O87,O88,O89,O 90,O91,O92,O94,O95,O98,O99,Z37

Available at: http://www.bccdc.ca/resource-gallery/Documents/Chronic-Disease-Dashboard/diabetes-mellitus.pdf

Exclusion codes for insulin dependent diabetes:

The PNET records of the previously identified DM population (either by CDR or through the BC-HTC algorithm) are evaluated. If any the following DINPIN codes is found in the PNET data of a given DM individual, then this person is flagged as insulin-dependent DM (else, of course, non-insulin-dependent DM)

PharmaNet Insulin DINPINs: (1934074, 1959220, 2024284, 586714, 2024233, 446564, 612227, 1934112, 612197, 632651, 632686, 1986085, 1986805, 1986813, 2025256, 1959239, 2024268, 587737,2024225, 446572, 612235, 1934066, 646148, 2024241, 446580, 612278, 1934090, 2024403, 2241310, 2415089, 2403447, 795879, 2024217, 1959212, 2025248, 889121, 2024446, 1962639, 2024292, 1962655, 2024306, 1962663, 2024322, 1962647, 2024314, 889091, 889105, 889113, 2466864, 644358, 733075, 2024276, 513644, 2275872,514551, 552275, 612170, 1985949, 2022249, 2275864, 514535, 612162, 612359, 2244353, 2245397, 2377209, 2460416, 2460424, 2460408, 2265435, 2265443, 2467879, 2467887, 2474875, 2412829, 2271842, 2245689, 2276410, 2251930, 2444844, 2294338, 2444852, 2461528, 2441829, 2493373, 2478293, 2279479, 2279460, 2279487,

2294346, 2229704, 2469901, 2229705, 2469898, 2233562, 2241283, 2403412, 2469871, 2439611, 2470152, 2240294, 2240295, 2403420, 2240297, 2403439, 632694, 650935, 1986821, 2022230, 552259, 614416, 1985957, 1985981, 612189, 552267, 628301, 723789, 1985930, 446610, 612219, 612200, 446599, 612243, 1934104, 446602, 612251, 1934082, 773654, 1985973, 632678, 1985965, 5894, 6009, 274119, 274127, 275409, 275417, 275425, 539201, 539244, 542911, 542938, 542946, 546348, 554820, 648094, 999717, 1986791, 45230001, 45230002, 45230003, 45230004, 45230005, 45230006, 45230007, 45230008, 45230009, 45230010, 45230011, 45230012, 45230013, 47450001, 47450002, 47450003, 47450004, 47450005, 47450006, 47450007, 66123203, 66124134, 66124135, 66124225, 66124582, 45230014, 45230015, 45230016, 46340034, 46340035, 46340036, 46340037,47450008, 47450009.

Reference:

1. Jeong D, Karim ME, Wong S, Wilton J, Butt ZA, Binka M, Adu P, Bartlett S, Pearce M, Clementi E, Yu A, Alvarez M, Samji, Velasquez H, Abdia Y, Krajden M, Janjua NZ. (2021).

Impact of HCV infection and ethnicity on incident type 2 diabetes: findings from a large population-based cohort in British Columbia. *BMJ Open Diabetes Research & Care*, 9(1):e002145, doi: 10.1136/bmjdrc-2021-002145

2. Chen G, Khan N, Walker R, et al. . Validating ICD coding algorithms for diabetes mellitus from administrative data. Diabetes Res Clin Pract 2010;89:189–95. 10.1016/j.diabres.2010.03.007

2) Variable: Diabetes mellitus screening

Data source : MSP; fee item codes: 91745, 91707, 91715, 91716

Definition: Diabetes Canada guidelines recommend that all individuals ≥40 years of age in the absence of diabetes risk factors, undergo diabetes screening every three years. Screening tests include either a fasting plasma glucose (FPG) or a Haemoglobin A1C (HbA1c). A 75g oral glucose tolerance test (75g-OGTT) can be used when diabetes or prediabetes is suspected. An individual was considered 'screened' if a fee item code was present for the measurement of HbA1C and/or FPG /or OGTT/or Random Plasma Glucose (RPG) at least 6 months apart.

Reference: 1) Diabetes Canada Clinical Practice Guidelines Expert C, Ekoe JM, Goldenberg R, Katz P. Screening for Diabetes in Adults. Canadian journal of diabetes. 2018;42 Suppl 1:S16-S9. Epub 2018/04/14. 2) British Columbia Ministry of Health [creator]. Medical Services Plan (MSP) Payment Information File. British Columbia Ministry of Health [publisher]. Data Extract. MOH (2021). https://www2.gov.bc.ca/gov/content/health/health-forms/online-services

3) Variable: Urban/Rural Residence

Data Source: Government of Canada/BC Ministry of Health/Client Roster

Definition: Community Health Service Areas (CHSA) Rural/urban classification enhances the ability to understand and monitor health needs and utilization patterns in CHSAs across the province. Following points were considered in the development of these 7-tier urban/rural classifications: statistical area classification type, population centre and rural area classification, and index of remoteness. Based on these, the following CHSA urban/rural designations were developed: Metropolitan, Large Urban, Medium Urban, Small Urban, Rural Hub, Rural and Remote.

Reference: Community Health Service Areas - CHSA - B.C. Health Region Master table - Version 2022. Available at:https://open.canada.ca/data/en/dataset/68f2f577-28a7-46b4-bca9-7e9770f2f357/resource/53f7c833-07a0-4d7b-ac59-f7c3125d7f34?inner_span=True
