# Supplementary material for: Impact of the COVID-19 Pandemic and Control Measures on Screening and Diagnoses of Type 2 Diabetes in British Columbia
Source: Int J Environ Res Public Health. 2025 Mar 28;22(4):519. doi: 10.3390/ijerph22040519 (PMC12026491; doi:10.3390/ijerph22040519)
Supplement: Supplementary file 1 [file ijerph-22-00519-s001.zip › Supplementary Tables_IJERPH_Revised_PR.pdf]

## Supplementary Tables

Table S1: Total number of BC adults (≥ 40 years ) eligible for screening and screened in 2016

| Population | Eligible for screening | Number Screened |
|---|---|---|
| All (N) | 6996936 | 930046 (13%) |
| Sex | | |
| Female | 3744368 | 504285 (54%) |
| Male | 3252561 | 425761 (46%) |
| Unknown | 7 | |
| Age Group | | |
| 40 - 49 | | 170342 |
| 50 - 59 | | 267771 |
| 60 - 69 | | 265955 |
| 70 - 79 | | 155951 |
| > 79 | | 70027 |
| Urban/Rural Residence | | |
| Metropolitan | | 417482 |
| Large Urban | | 167686 |
| Medium Urban | | 93434 |
| Small Urban | | 88024 |
| Rural Hub | | 49349 |
| Rural | | 105795 |
| Remote | | 7567 |
| Missing | | 709 |
| Health Authority | | |
| Fraser | | 310575 |
| Interior | | 158204 |
| Northern | | 49406 |
| Vancouver Coastal | | 229827 |
| Vancouver Island | | 181325 |
| Missing | | 709 |

Table S2: Total number of BC adults ≥18 years diagnosed with diabetes in 2016

| Population | ≥18 years in 2016 | Number of individuals diagnosed |
|---|---|---|
| **Total (N)** | 9488922 | 37115 (.04%) |
| **Sex** | | |
| Female | 5150330 | 17,216 (46%) |
| Male | 4338583 | 19,899 (54%) |
| Unknown | 9 | |
| **Age Group** | | |
| 18-29 | | 964 |
| 30-39 | | 2,859 |
| 40 - 49 | | 6,068 |
| 50 - 59 | | 10,054 |
| 60 - 69 | | 9,972 |
| 70 - 79 | | 5,191 |
| > 79 | | 2,007 |
| **Urban/Rural Residence** | | |
| Metropolitan | | 19,647 |
| Large Urban | | 5,809 |
| Medium Urban | | 3,595 |
| Small Urban | | 3,099 |
| Rural Hub | | 1,594 |
| Rural | | 3,001 |
| Remote | | 273 |
| Missing | | 97 |
| **Health Authority** | | |
| Fraser | | 15,597 |
| Interior | | 5,301 |
| Northern | | 2,071 |
| Vancouver Coastal | | 8,643 |
| Vancouver Island | | 5,406 |
| Missing | | 97 |

Table S3: Difference in number of diabetes screening following pandemic-related policies, compared to counterfactual, by period, age group and sex

| **Population** | | **Absolute difference, n (95% CI)** | | | **Percentage difference, % (95% CI)** | | |
|---|---|---|---|---|---|---|---|
| Age-group | Sex | Apr 1st 2020 - Dec 31st 2020 | Jan 1st 2021- Dec 31st 2021 | Jan 1st 2022 - Dec 31st 2022 | Apr 1st 2020 - Dec 31st 2020 | Jan 1st 2021- Dec 31st 2021 | Jan 1st 2022 – Dec 31st 2022 |
| 40 - 49 | Females | -11292(-14609,-7981) | -3486(-8331,1225) | -8793(-13874,-3951) | -24.3(-30.3,-18.0) | -6.0(-13.9,2.3) | -17.2(-25.3,-8.4) |
| | Males | -11084(-13397,-8773) | -6603(-10052,-3241) | -9796(-13529,-6224) | -30.3(-25.3,-25.0) | -14.4(-20.9,-7.5) | -23.5(-30.4,-6.1) |
| 50 - 59 | Females | -20507(-28500,-12552) | 7123(-5756,19754) | 9258(-6126,24032) | -18.9(-25.3,-12.1) | 5.0(-3.8,14.4) | 6.6(-3.9,18.0) |
| | Males | -21258(-26975,-15545) | -3753(-13204,5491) | 907(-10531,11904) | -23.0(-28.2,-17.4) | -2.9(-9.9,4.6) | 0.8(07.8,10.0) |
| 60 - 69 | Females | -19627(-28367,10930) | 5759(-8205,19440) | 11939(-5300,28587) | -16.9(-23.6,-9.9) | 3.8(-5.0,13.1) | 7.6(-3.0,19.2) |
| | Males | -19076(-26034,-12222) | -2542(-13793,8452) | 4937(-9100,18429) | -18.2(-24.0,-12.1) | -1.7(-9.0,6.1) | 3.5( -5.7,13.3) |
| 70 - 79 | Females | -12535(-19231,-5884) | -195(-11353,10625) | 4083(-10293,17719) | -15.5(-22.9,-7.5) | 0.01(-9.2,9.9) | 3.6(-7.6,15.9) |
| | Males | -11638(-17151,-6205) | -3596(-12609,5197) | -1032(-12738,10214) | -15.7(-22.3,-8.8) | -3.3(-11.2,5.2) | -0.7(-10.3,9.6) |
| > 79 | Females | 5026(-8185,-1867) | 1818(-3474,6962) | 5422(-1334,11891) | -13.9(-21.8,-5.5) | 3.7(-6.3,14.5) | 10.2(-2.3,23.9) |
| | Males | -3337(-5626,-1062) | 934(-2903,4674) | 3504(-1399,8225) | -11.7(-19.0,-3.8) | 2.4(-6.6,12.1) | 8.3(-3.0,20.6) |

Table S4: Difference in number of diabetes diagnoses following pandemic-related policies, compared to counterfactual, by period, age group and sex.

| **Population** | | **Absolute difference, n (95% CI)** | | | **Percentage difference, % (95% CI)** | | |
|---|---|---|---|---|---|---|---|
| Age-group | Sex | Apr 1st 2020 - Dec 31st 2020 | Jan 1st 2021- Dec 31st 2021 | Jan 1st 2022 - Dec 31st 2022 | Apr 1st 2020 - Dec 31st 2020 | Jan 1st 2021- Dec 31st 2021 | Jan 1st 2022 – Dec 31st 2022 |
| 18 - 29 | Females | 71(22,119) | 129(67,187) | 165(89,237) | 26.9(7.7,48.7) | 39.7(17.6,64.6) | 56.4(25.1,92.9) |
| | Males | 26(-18,71) | 61(1,117) | 34(-31,94) | 14.3(8.5,41.1) | 27.8(0.5,59.9) | 18.4(-11.9,55.5) |
| 30 - 39 | Females | 58(-45,161) | 400(248,548) | 537(358,709) | 6.6(-4.9,19.0) | 35.3(20.0,52.3) | 49.7(29.4,72.4) |
| | Males | -3(-92,85) | 229(98,355) | 226(74,369) | -0.3(-11.0,11.4) | 22.7(8.8,38.0) | 23.8(6.9,42.4) |
| 40 - 49 | Females | 15(-143,174) | 704(466,936) | 1009(738,1275) | 1.2(-9.0,12.3) | 38.0(23.0,54.1) | 58.7(38.2,81.3) |
| | Males | -89(-235,56) | 456(241,668) | 503(265,735) | -5.3(-13.6,3.5) | 21.9(10.7,33.9) | 26.2(12.5,41.3) |
| 50 - 59 | Females | -87(-316,142) | 969(621,1311) | 1371(980,1756) | -3.5(-12.3,6.1) | 31.9(19.0,45.8) | 48.7(31.5,67.5) |
| | Males | -2919-525,-58) | 648(294,992) | 852(459,1230) | -10.5(-18.3,-2.2) | 19.0(8.1,30.7) | 27.3(13.3,42.5) |
| 60 - 69 | Females | -169(-383,45) | 827(503,1146) | 908(551,1256) | -6.8(-14.9,2.0) | 27.3(15.4,39.9) | 32.3(17.7,48.1) |
| | Males | -201(-463,62) | 606(213,988) | 951(507,1377) | -7.0(-15.6,2.3) | 17.1(5.6,29.5) | 28.9(14.0,45.4) |
| 70 - 79 | Females | -129(-283,26) | 450(208,686) | 682(397,953) | -8.6(-18.0,1.7) | 23.7(10.0,38.8) | 37.3(19.2,57.5) |
| | Males | -162(-225,12) | 345(78,607) | 547(232,846) | -9.2(-18.4,0.7) | 15.6(3.2,29.3) | 25.9(9.8,43.8) |
| > 79 | Females | -72(-149,5) | 114(-3,227) | 216(74,349) | -10.7(-21.1,0.7) | 13.6(-0.3,28.8) | 26.1(7.8,46.7) |
| | Males | -63(-146,21) | 123(-6,247) | 208(49,357) | -9.2(-20.5,3.2) | 14.2(-0.7,30.6) | 23.2(4.9,26.0) |

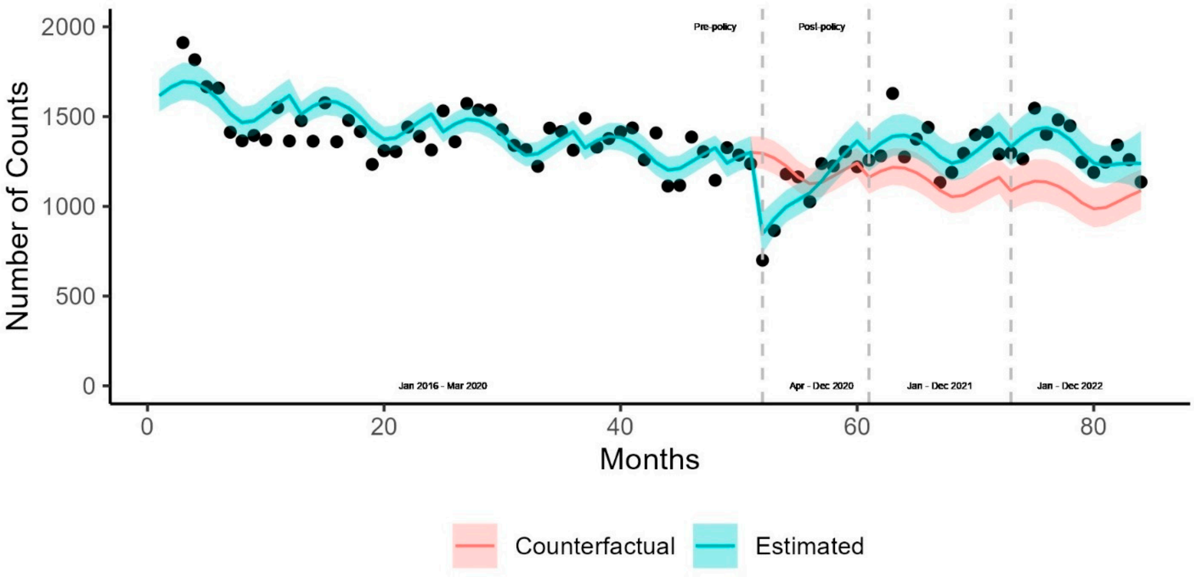


Figure S1: Diabetes diagnoses males (all ages).

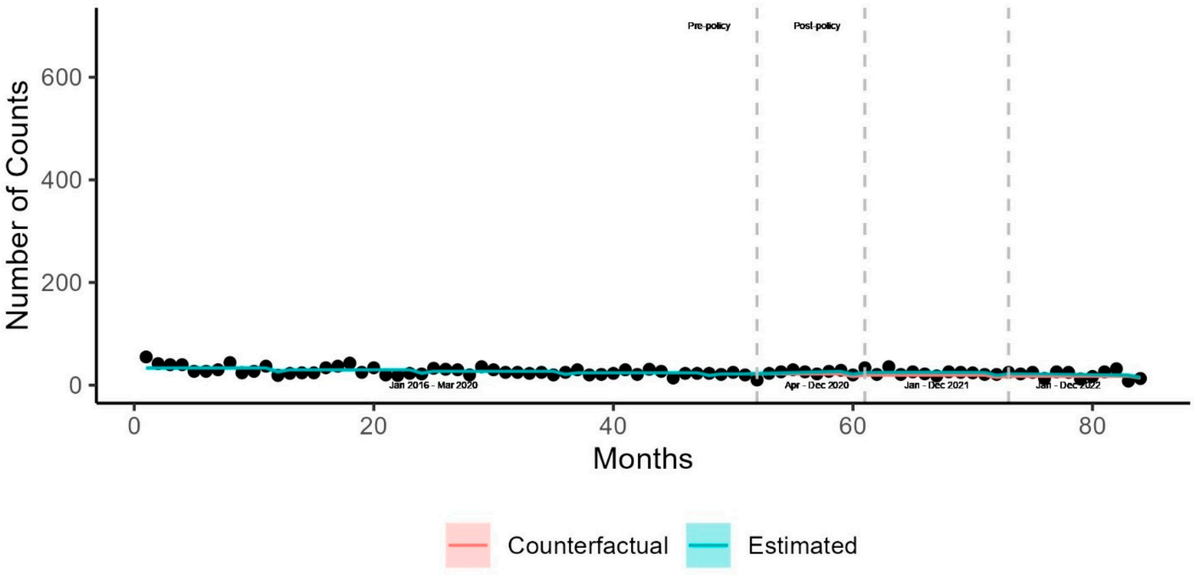


Figure S2: Diabetes diagnoses males (18 -29 years).

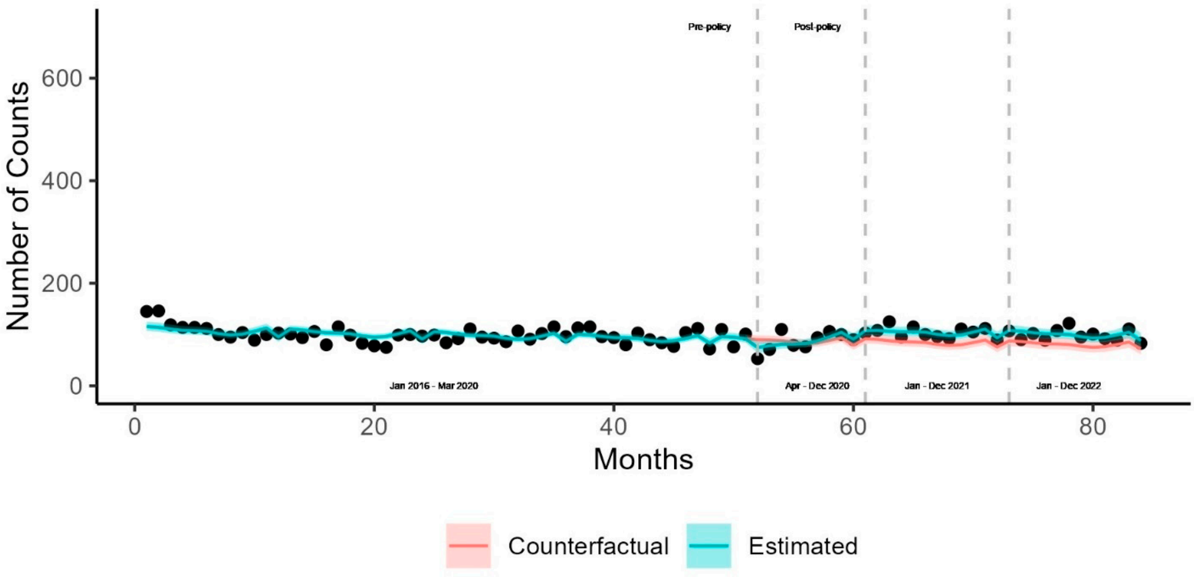


Figure S3: Diabetes diagnoses males (30-39 years).

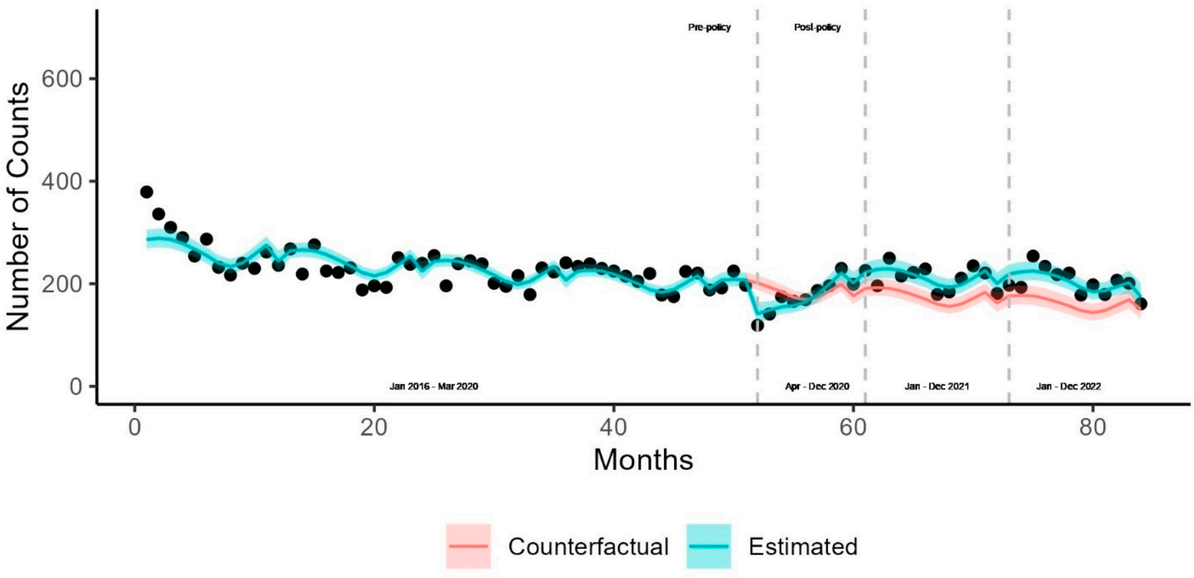


Figure S4: Diabetes diagnoses males (40-49 years).

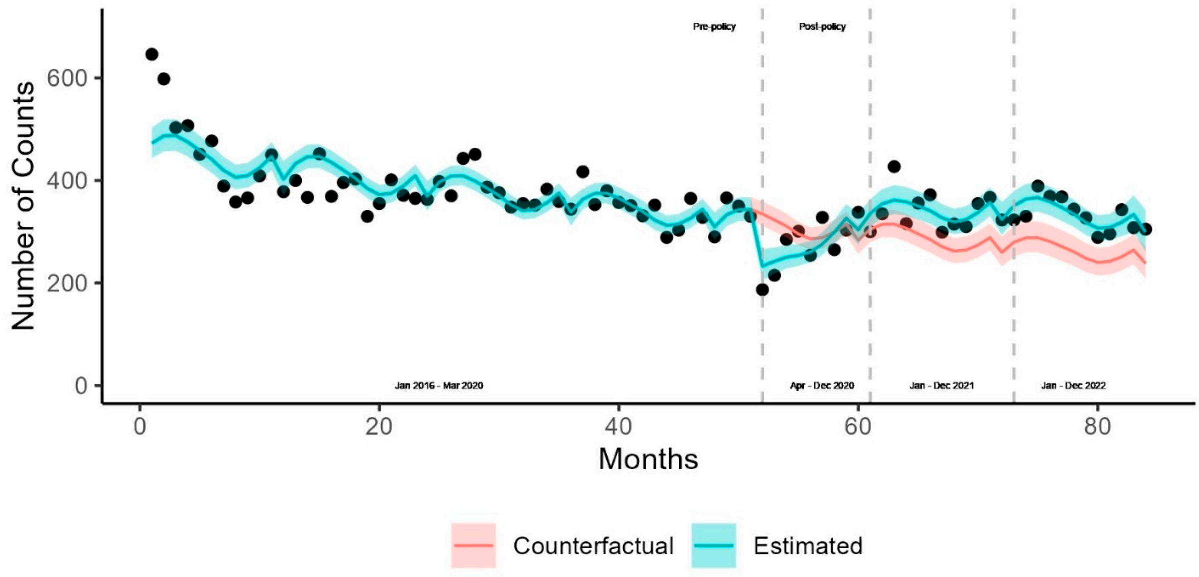


Figure S5: Diabetes diagnoses males (50-59 years).

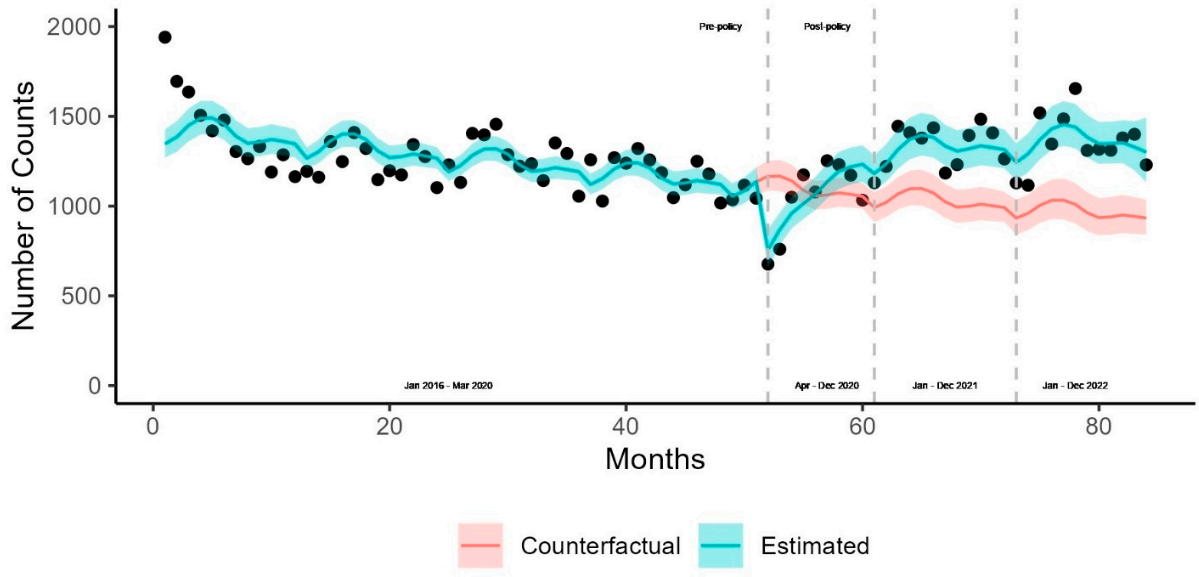


Figure S6: Diabetes diagnoses females (all age groups).

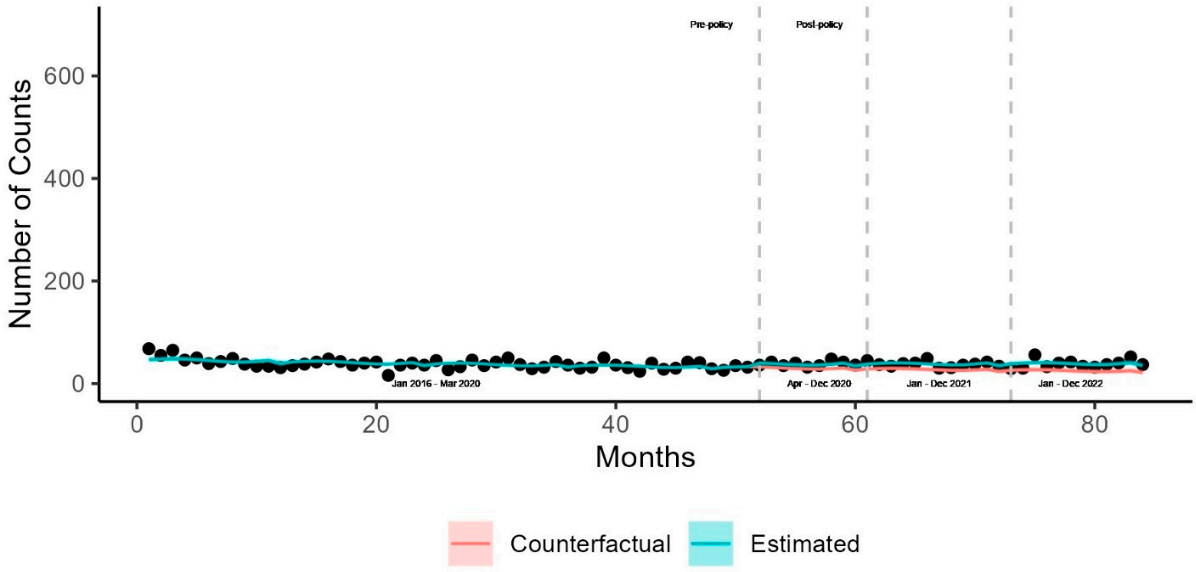


Figure S7: Diabetes diagnoses females (18-29 years).

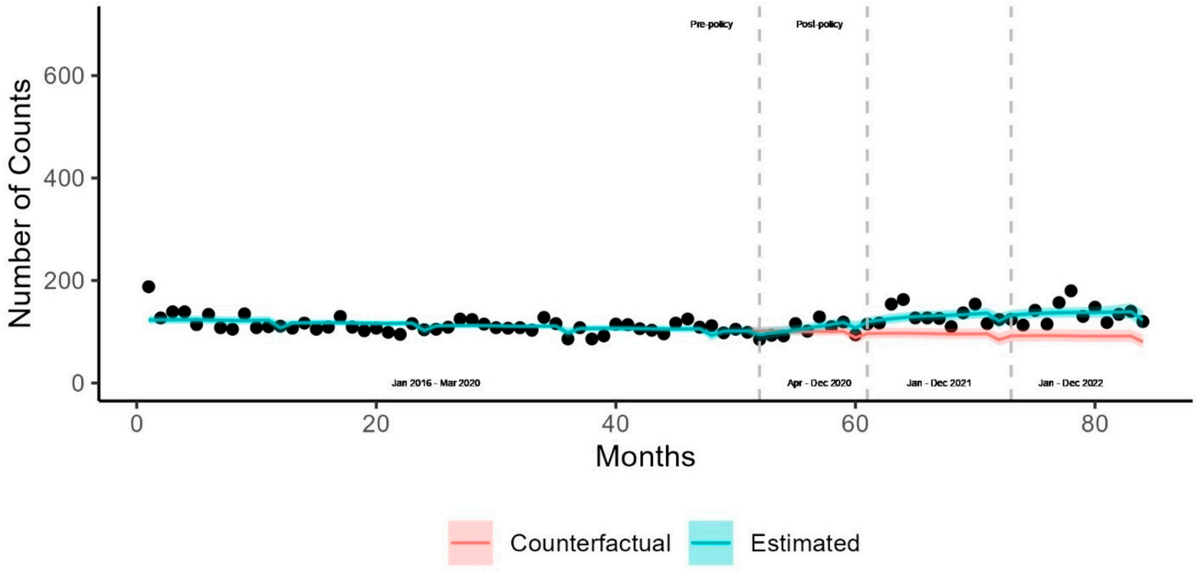


Figure S8: Diabetes diagnoses females (30-39 years).

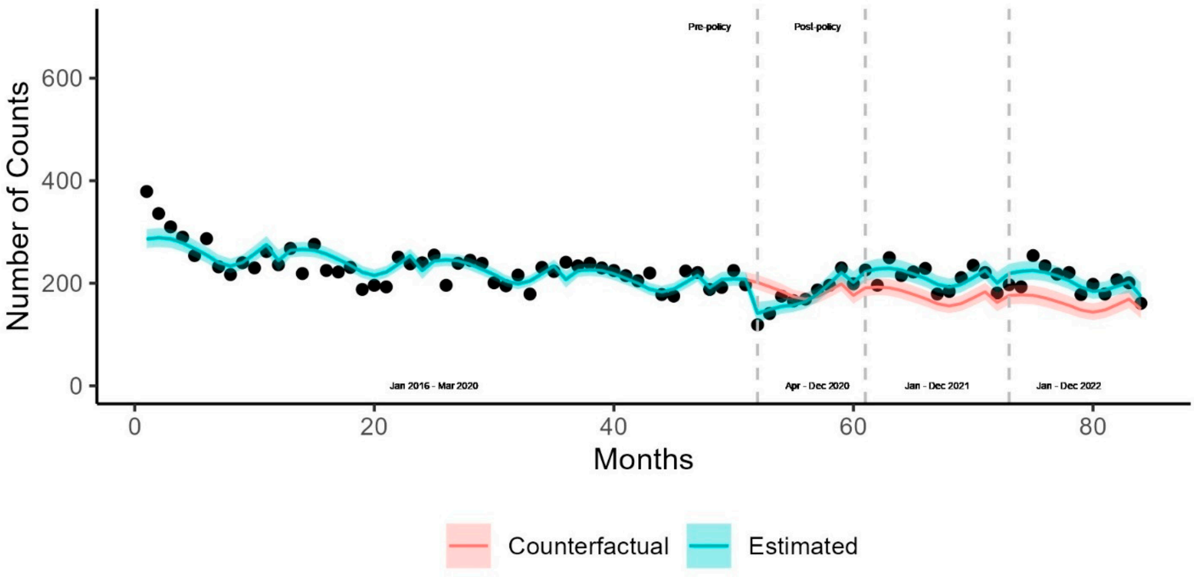


Figure S9: Diabetes diagnoses females (40-49 years).

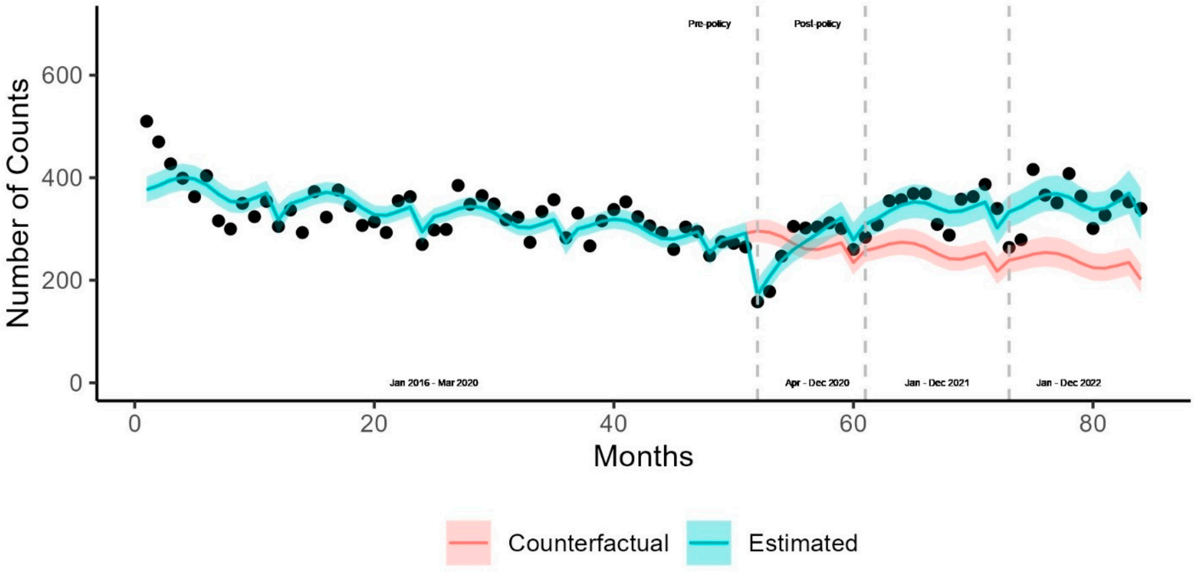


Figure S10: Diabetes diagnoses females (50-59 years).
